# Proteomics validate circulating GDF-15 as an independent biomarker for COVID-19 severity

**DOI:** 10.3389/fimmu.2024.1377126

**Published:** 2024-04-15

**Authors:** Simeng Bu, Léna Royston, Tsoarello Mabanga, Carolina A. Berini, Cécile Tremblay, Bertrand Lebouché, Joseph Cox, Cecilia T. Costiniuk, Madeleine Durand, Stephane Isnard, Jean-Pierre Routy

**Affiliations:** ^1^ Research Institute of the McGill University Health Centre, Montreal, QC, Canada; ^2^ Chronic Viral Illness Service, McGill University Health Centre, Montreal, QC, Canada; ^3^ Division of Infectious Diseases, Geneva University Hospitals, Geneva, Switzerland; ^4^ Département de Microbiologie, Infectiologie et Immunologie, Université de Montréal, Montréal, QC, Canada; ^5^ Département de Microbiologie, Infectiologie et Immunologie, Centre de Recherche du Centre Hospitalier de l’Université de Montréal, Montréal, QC, Canada; ^6^ Division of Hematology, McGill University Health Centre, Montreal, QC, Canada

**Keywords:** GDF-15, COVID-19, SARS-CoV-2, proteomics, BQC19, severity

## Abstract

**Introduction:**

Growth differentiation factor 15 (GDF-15) was originally described as a stress-induced cytokine, and a biomarker of aging and cardiovascular diseases. We hypothesized that circulating GDF-15 would be associated with COVID-19 disease severity. Herein, we explored this hypothesis in a large cohort of COVID-19 patients.

**Methods:**

Blood samples were collected from 926 COVID-19 adult patients and from 285 hospitalized controls from the Biobanque Québécoise de la COVID-19 (BQC19). COVID-19 severity was graded according to the WHO criteria. SOMAscan proteomics assay was performed on 50µL of plasma. ELISA were performed on 46 selected participants with left-over plasma to validate differences in plasma GDF-15 levels. Statistical analyses were conducted using GraphPad Prism 9.0 and SPSS. P values < 0.01 were considered significant.

**Results:**

Proteomics showed that plasma GDF-15 levels were higher in COVID-19 patients compared to hospitalized controls. GDF-15 levels increased with COVID-19 severity. COVID-19 patients presenting with comorbidities including diabetes, cancer, chronic obstructive pulmonary disease (COPD) and cardiovascular disease had higher GDF-15 levels. ELISA revealed significant elevation of GDF-15 until 30 days after hospitalization. Plasma GDF-15 elevation was correlated with older age. Moreover, GDF-15 levels correlated with pro-inflammatory cytokine interleukin-6 (IL-6) and inflammation marker C-reactive protein (CRP) as well as soluble levels of its putative receptor CD48. No association was established between anti-SARS-CoV-2 IgG levels and plasma GDF-15 levels.

**Conclusions:**

This study confirms GDF-15 as a biomarker for COVID-19 severity. Clinical evaluation of GDF-15 levels could assist identification of persons at high-risk of progressing to severe disease, thus improving patient care.

## Introduction

Coronavirus disease-2019 (COVID-19) is a highly contagious infectious condition caused by severe acute respiratory syndrome coronavirus 2 (SARS-COV-2) (1). The clinical manifestations of COVID-19 are heterogeneous across populations. Numerous studies underscore the connection between mortality from COVID-19 and underlying comorbidities including obesity, diabetes, chronic obstructive pulmonary disease (COPD), and cardiovascular diseases (CVD) (2–5). Moreover, adverse outcomes have also been observed in COVID-19 infected individuals undergoing chemotherapy and chemotherapy or immunotherapy for cancer treatment (6). The progression to a severe and fatal stage of the disease can occur rapidly and unexpectedly. Thus, the identification of prognostic or diagnostic biomarkers for the severity of COVID-19 is imperative to improve patient management.

Growth differentiation factor 15 (GDF-15) belongs to the transforming growth factor-β (TGF-β) superfamily and is recognized as a novel marker for aging, weight and appetite modulation (7–9). Several investigations have linked elevated GDF-15 levels in the bloodstream to various age-related conditions, including cardiovascular diseases and diabetes, including in people living with HIV (PLWH) (10–12). GDF-15 levels are also elevated in patients with certain types of advanced cancers, although conflicting findings have been described on the role of GDF-15 during early or late tumorigenesis (13–15). Teng et al. reported an association between GDF-15 and severity of COVID-19, correlated with a poorer clinical outcome and SARS-CoV-2 viremia in 78 COVID-19+ participants (16). Similarly, Alserawan et al. found that GDF-15 levels were correlated with well-established pro-inflammatory markers including IL-6, CRP, ferritin and D-dimer, and served as a biomarker for lung impairment in 84 patients with COVID-19 (17). However, these studies did not establish the independent prognostic value of GDF15 in COVID-19 as several comorbidities are also associated with elevated GDF15 levels.

We aimed to confirm and expand previous research findings and validate the prognostic value of GDF-15 as a marker for COVID-19 severity in a large, more diverse population with a range of comorbidities, in hospitalized patients before the vaccination era. Using the *Biobanque Québécoise de la COVID-19* (BQC19) (18) and plasma proteomics analysis, we quantified GDF-15 and other markers of interest for a total of 1211 participants, representing both COVID-19 positive cases and non-COVID-19 hospitalized controls.

## Methods

### Participant and sample collection

BQC19 is a provincial-wide biobank established in March 2020 to enable collection, storage, and sharing of samples and data of people affected by COVID-19 disease in Quebec, Canada (see bqc19.ca) (18).

Briefly, participants were recruited at the hospital and invited to participate between March 2020 and August 2021. Patients with SARS-CoV-2 diagnosis validated by RT-PCR or disease presentation were included. Medical history and clinical data were obtained from questionnaires and medical charts, and blood was collected at several timepoints.

After selecting for data from adult participants, we considered samples, clinical and proteomics data from 926 adults diagnosed with COVID-19 and 285 hospitalized controls. COVID-19 was diagnosed using RT-qPCR testing. All controls had negative COVID-19 tests. Controls were hospitalized for various reasons such as infectious respiratory symptoms (not linked to COVID-19), acute cardiovascular events, emboly, digestive symptoms not requiring surgery, uncontrolled diabetes. For participants with multiple blood samples collected during hospitalization, the samples collected at the earliest time point related to symptom onset were used to reflect the proteome of acute COVID-19, and to avoid use of measures during the recovery phase. No participant had received COVID-19 vaccination at the time samples were collected.

COVID-19 participants were categorized into mild, moderate or severe infections based on criteria from the WHO Working Group on the Clinical Characterisation and Management of COVID-19 (19). Briefly, mild or ambulatory disease included patients with asymptomatic presentation while having detectable SARS-CoV-2, or symptomatic independent, or requiring low assistance. Moderate diseases encompass hospitalized patients not requiring oxygen therapy or requiring oxygen by mask or nasal prongs. Severe disease refers to hospitalized patients requiring oxygen, intubation or mechanical ventilation, dialysis or ECMO. Participants who died during their hospitalization were included in the “fatal” infection group.

A total of 15 recovered COVID-19 participants were recruited at the McGill University Health Centre. Inclusion criteria included being adult, having received a diagnostic of COVID-19 at more than 14 days ago, and not having COVID-19 symptoms. People presenting with long-COVID-19 symptoms were excluded. Sample from controls were collected before December 2019 in Quebec, Canada, or from January 2020 to January 2021, with no history of COVID-19 tests or symptoms.

### Blood samples preparation

Whole blood was obtained through venipuncture using acid-citrate-dextrose vacutainer tubes or EDTA tubes for recovered participants, and plasma was separated by centrifugation at 750g, 10 min at room temperature. Isolated plasma was aliquoted and stored at −80°C until analysis.

### Proteomic measurement using the SOMAscan platform

Blood samples from a total of 1211 BQC19 participants were included for the plasma proteomics analysis. Proteomic profiles were assessed at SomaLogic using the SomaScan v4.0 proteomic platform that provides measurements on 4701 unique human circulating proteins using 4987 Slow Off-Rate Modified Aptamers (SOMAmer reagents) and quantifies protein levels in the form of relative fluorescence units (RFUs) (20). Experimental process and data normalization including hybridization control normalization, intraplate median signal normalization, and plate scaling and calibration were performed as previously described (20, 21).

### Measurement of plasma GDF-15 levels using ELISA

When samples were available, plasma levels of human GDF-15 were measured in duplicates using the enzyme-linked immunosorbent assay (ELISA) assay (R&D systems, MN, USA) as per the manufacturer’s instructions.

### Measurement of spike specific IgG levels using cell-based ELISA

Spike-specific IgG levels were quantified using a cell-based ELISA (CBE) method as previously described (22). Briefly, SARS-CoV-2 Spike-expressing HOS cells were washed and incubated with diluted plasma (1:250). After wash, anti-human IgG antibody coupled to horseradish peroxidase (HRP) were incubated. After wash and substrate addition, light emission was detected using a luminometer.

### Statistical analysis

Statistical analyses were conducted using GraphPad Prism 9.0 (GraphPad, CA, USA). Spearman’s rank correlation test identified associations between 2 continuous variables. Mann-Whitney U test and student t-test were used to compare levels of continuous variables between two independent groups, as appropriate. Kruskal-Wallis one way ANOVA test was used to compare levels of continuous variables in more than 2 independent study groups. Paired analysis was performed by Wilcoxon signed-rank test. P-values < 0.001 were considered significant for samples with n>250, and p<0.05 for samples less<250. Logistic regression univariable models were used to generate Receiver operating characteristic (ROC) curve. Multivariable analysis was performed using SPSS.

### Ethics

BQC19 received ethical approval from the institutional review board (IRB) of the Jewish General Hospital and the Centre Hospitalier de l’Université de Montréal (CHUM) in Montréal, QC, Canada. All participants gave informed consent. Secondary analysis of BQC19 data and additional analyses for this project were approved by the research ethics board of the McGill University Health Centre (MUHC).

## Results

### Participants characteristics and clinical outcomes

The demographic and clinical characteristics of all study participants are presented in **Table 1**. In total, there were 926 RT-qPCR positive COVID-19 cases (median age: 60; range 18-99), encompassing 46% female and 54% male. In addition, 285 RT-qPCR COVID-19 negative hospitalized participants were included as controls (median age 56, range 20-99): 47% were female and 53% were male. In the RT-qPCR confirmed COVID-19 positive cohort (n=926), 256 were classified as mild, 384 as moderate, 235 as severe and 24 as fatal using WHO criteria. Clinical outcomes and comorbidities were extracted from clinical charts and assessed for all participants including those who had died.

**Table 1:** Characteristics of all proteomics study participants.

COVID-19 Severity	COVID-19 positive (n=926)	COVID-19 negative (n=285)
**Age** **Range**	60 (18-99)	56 (20-99)
**Sex: Women** **Men**	428 (46.22%) 498 (53.78%)	135 (47.37%) 150 (52.63%)
COVID-19 Severity		
Mild	256	
Moderate	384	
Severe	235	N/A
Dead	24	
n/a	27	
Obesity		
No	822 (88.77%)	261 (91.58%)
Yes	91 (9.83%)	23 (8.07%)
Missing	13 (1.40%)	1 (0.35%)
Diabetes		
No	670 (72.36%)	223 (78.25%)
Yes	247 (26.67%)	61 (21.40%)
Missing	9 (0.97%)	1 (0.35%)
HIV		
No	906 (97.84%)	282 (98.95%)
Yes	7 (0.76%)	2 (0.70%)
Missing	13 (1.40%)	1 (0.35%)
Cancer		
No	814 (87.90%)	238 (83.51%)
Yes	105 (11.34%)	46 (16.14%)
Missing	7 (0.76%)	1 (0.35%)
Chronic obstructive pulmonary disease		
No	832 (89.85%)	262 (91.93%)
Yes	84 (9.07%)	22 (7.72%)
Missing	10 (1.08%)	1 (0.35%)
Cardiovascular disease		
No	438 (47.30%)	212 (74.39%)
Yes	488 (52.70%)	72 (25.26%)
Missing	n/a	1 (0.35%)

### Proteomics identifies higher plasma GDF-15 levels in severe COVID-19

**Figure 1:**
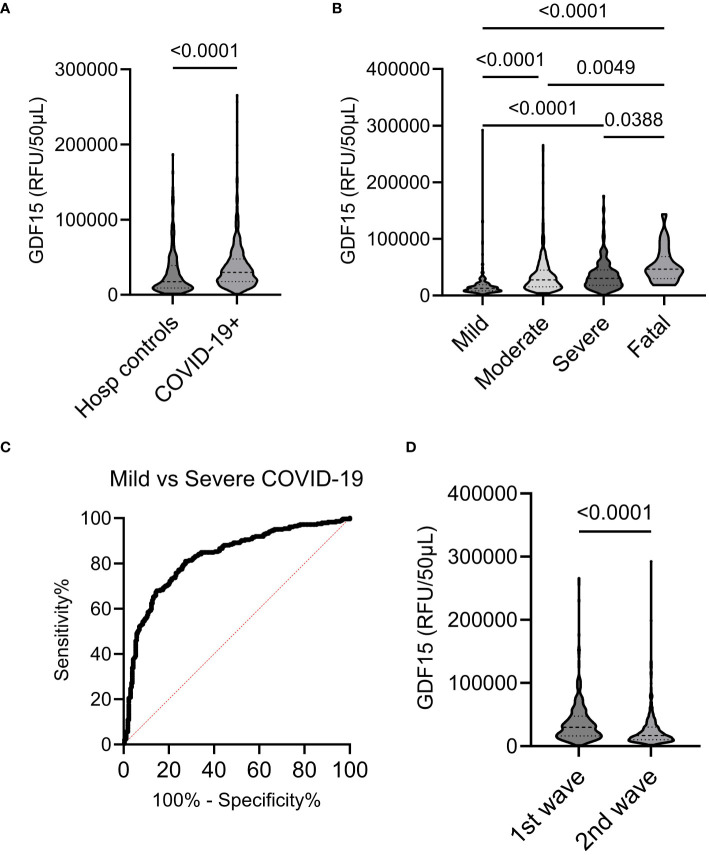
Plasma GDF-15 in COVID-19 groups and controls. **(A, B)** Violin plots showing plasma GDF-15 levels in COVID-19 infected and hospitalized controls and among different COVID-19 severity groups. **(C)** Plasma levels of GDF-15 in mild and severe COVID-19 groups were evaluated by ROC curve. **(D)** Plasma levels of GDF-15 in 2020 and 2021.

Proteomics assay measured plasma protein levels in all COVID-19 infected and control adult participants. Plasma GDF-15 levels were significantly higher by 1.7-fold in COVID-19 infected participants compared to controls (p<0.0001) (**Figure 1A**). In COVID-19 positive patients, as COVID-19 severity worsened from mild to fatal, the plasma GDF-15 levels increased with the highest median GDF-15 levels detected in the fatal group (p<0.0001) (**Figure 1B**). Severe COVID-19 groups also had significantly greater plasma GDF-15 elevation compared to moderate and mild COVID-19 groups (p=0.0049, p<0.0001 respectively). Multivariable analysis showed that age, but not sex nor presence of comorbidities, was a confounding factor of the association between GDF-15 and disease severity (**Table 2**), because GDF-15 was associated with age (Manova p < 0.0001) but not Severity (Manova p = 0.89) (**Table 2**) Age-adjusted regression analysis increased association between plasma GDF-15 and COVID-19 severity (unadjusted β: 6.10^6^, adjusted β: 0.222). ROC analysis was conducted to evaluate the predictive ability of plasma GDF-15 to discriminate mild and severe COVID-19 states (including fatal cases) (**Figure 1C**). The area under the curve (AUC) was estimated for observed GDF-15 levels and their predicted values by fitting regression models. Observed GDF-15 levels were able to predict COVID-19 disease severity (AUC=0.82 ± 0.02, p<0.0001). These results support the potential of plasma GDF-15 levels in predicting COVID-19 severity.

**Table 2:** Characteristics of ELISA GDF-15 study participants.

COVID-19 Severity	Controls (n=21)	Acute (n=28)	Early Recovered (n=10)	Late Recovered (n=19)
**Age**	40 (21-63)	60 (35-92)	58 (30-88)	43 (20-70)
**Recovery Duration**	N/A	2 (0-2)	30 (30-61)	223 (99-406)
**Sex: Women** **Men**	6 15	7 7	6 2	11 8

Plasma levels of other 5200 markers were compared in patients with mild and severe/fatal disease presentation. GDF-15 was found as one of the 461 proteins associated with severity, with the most significant q and p value (-log_10_ 118.2; p<0.00001, **Supplementary Figure 1**). Q value was less significant for IL-6 and CRP (-log_10_ 35.81 and 108.3, respectively, p<0.00001 for both), two common biomarkers of severity.

### Plasma GDF-15 levels differed between the first and second wave of COVID-19 in Quebec, Canada

We compared plasma GDF-15 levels between the initial wave of COVID-19, spanning from March to July 2020, to those of the second wave from august 2020 to August 2021 in the Province of Quebec. Patients in both waves were mostly affected by the ancestral SARS-CoV-2 strains in Quebec. No participants received priori vaccination even though vaccination campaign started in 2021 in Quebec. Plasma levels of GDF-15 were notably higher during the first wave compared to those observed during the second COVID-19 wave (p<0.001) (see **Figure 1D**). The variation was independent of comorbidities, and sex in the two collection periods. However, the age of the patients during the second wave was lower than the age of patients in the first wave (57.4 vs 66.9, p<0.001).

### Higher GDF-15 levels in severe COVID-19 patients independently of comorbidities

**Figure 2:**
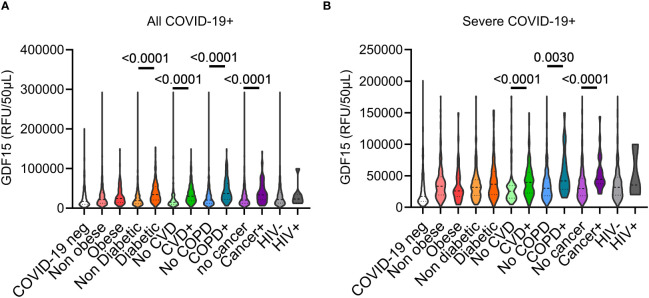
Expression patterns of plasma GDF-15 levels in different comorbidities in COVID-19 infected patients. **(A)** In all COVID-19 infected patients, higher plasma GDF-15 levels are observed in all comorbid groups relative to their non-comorbid conditions. Differences of GDF-15 expression was significant in diabetic (p <0.001), cancer (p <0.001), COPD (p <0.001) and CVD (p <0.001) comorbidity groups relative to their non-comorbid conditions. **(B)** In severe and fatal COVID-19 patients, plasma GDF-15 levels are significantly higher in cancer (p <0.001), COPD (p <0.001) and CVD (p <0.001) comorbid groups.

A total of 926 COVID-19 infected patients were included in this study. The most common comorbidities were cardiovascular disease (CVD) (n=488, 52.70%), diabetes (247, 26.67%), cancer (105, 11.34%), obesity (91, 9.83%), COPD (84, 9.07%) and HIV (7; 0.76%) (**Table 3**). Compared to hospitalized controls, COVID-19 infected patients exhibited higher average plasma GDF-15 levels regardless of presence or absence of comorbidities. In all COVID-19 infected patients, GDF-15 levels were consistently higher in those with comorbidities groups. Diabetes, CVD, COPD, and cancer were the conditions significantly associated with higher GDF-15 levels (p<0.0001 for the three comparisons) (**Figure 2A**). Although higher levels of GDF-15 were noticed in obese (body mass index greater than 30) patients with COVID-19, this difference was not significant (p = 0.24). Studies have found a higher risk of hospitalization in Canadian PLWH (23, 24). We found no difference in GDF-15 levels between PLWH and HIV-negative patients, although the number of PLWH was small (n=7). Such observations indicate that elevated plasma GDF-15 levels in COVID-19 appears independent of patient’s comorbidities. We next examined the impact of these comorbidities on GDF-15 expression in severe and fatal COVID-19 patients. GDF-15 levels in these groups remained higher than that of hospitalized controls. Significantly higher levels of GDF-15 expression were observed in patients with CVD, COPD and cancer (p<0.0001, 0.0030 and <0.001 respectively) compared to severe cases without those conditions. Differences in obese or diabetic participants were not significantly different (**Figure 2B**). Multivariable analysis showed that increased levels of GDF-15 in patient with severe *vs* mild and moderate disease was independently associated with severity while adjusting for the presence of any comorbidity, sex and but was influenced by age (**Table 4**).

**Table 3:** Characteristics of COVID-19 positive proteomics participants.

COVID-19 Severity	Mild (n=256)	Moderate (n=384)	Severe (n=235)	Dead (n=24)
**Age**	55 (19-99)	63 (19-99)	65 (23-98)	71 (48-99)
**Sex: Women** **Men**	147 109	187 197	69 166	10 14
Obesity				
No	246 (96.10%)	351 (91.41%)	186 (79.15%)	20 (83.33%)
Yes	9 (3.52%)	31 (10.92%)	46 (19.57%)	3 (12.50%)
Missing	1 (0.39%)	2 (0.52%)	3 (1.28%)	1 (4.17%)
Diabetes				
No	231 (90.23%)	266 (69.27%)	138 (58.72%)	16 (66.67%)
Yes	24 (9.38%)	117 (30.47%)	95 (40.43%)	8 (33.33%)
Missing	1 (0.39%)	1 (0.26%)	2 (0.85%)	n/a
HIV				
No	251 (98.05%)	379 (98.70%)	231 (98.30%)	23 (95.83%)
Yes	2 (0.78%)	2 (0.52%)	2 (0.85%)	1 (4.17%)
Missing	3 (1.17%)	3 (0.78%)	2 (0.85%)	n/a
Cancer				
No	239 (93.00%)	323 (84.11%)	214 (91.06%)	19 (79.17%)
Yes	17 (6.61%)	60 (15.63%)	21 (8.94%)	5 (20.83%)
Missing	1 (0.39%)	1 (0.26%)	n/a	n/a
Chronic obstructive pulmonary disease				
No	243 (94.92%)	337 (87.76%)	210 (89.36%)	20 (83.33%)
Yes	12 (4.69%)	46 (11.98%)	22 (9.36%)	4 (16.67%)
Missing	1 (0.39%)	1 (0.26%)	3 (1.28%)	n/a
Cardiovascular disease				
No	170 (66.40%)	152 (39.58%)	87 (37.02%)	7 (29.17%)
Yes	86 (33.60%)	232 (60.42%)	148 (62.98%)	17 (70.83%)
Missing	n/a	n/a	n/a	n/a

**Table 4:** Multivariable correlations.

		Manova		
		Wilks’ delta	F value	P value
Plasma GDF-15 levels and COVID-19 severity	Age	**0.031**	1.342	**0.001**
	Sex	0.954	3.514	0.032
	Type of comorbidity	0.982	1.364	0.259

MANOVA test performed using SPSS with data from 900 participants with severity data. P value < 0.001 was considered significant.

Statistically significant differences were indicated in bold.

### ELISA confirms higher GDF-15 levels during COVID-19 and after recovery

**Figure 3:**
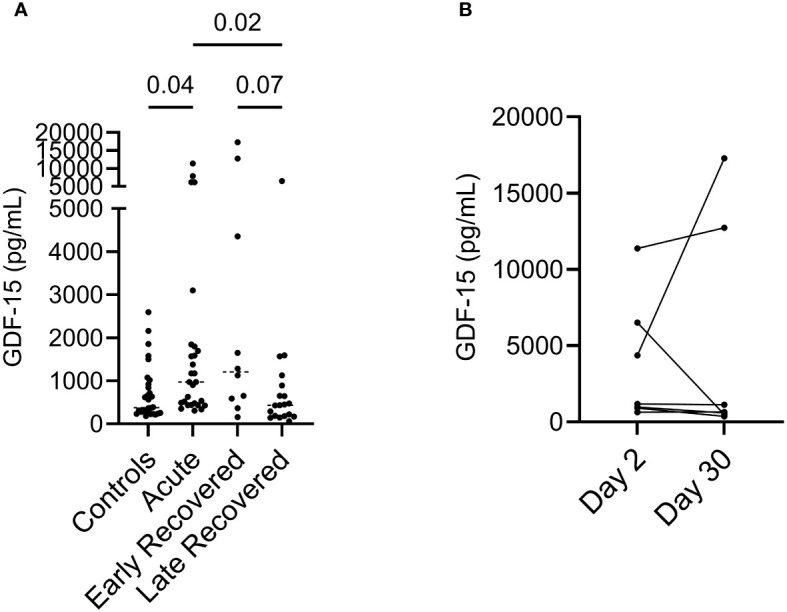
Elevated plasma GDF-15 levels in acute and early recovered COVID-19 infected patients. **(A)** ELISA results showed variations of GDF-15 levels among different COVID-19 cohorts. Kruskal-Wallis’s analysis showed that GDF-15 levels in acute cohorts are significantly higher than controls and late recovered cohorts. **(B)** M Wilcoxon paired analysis showed variations of plasma GDF-15 levels in 7 pairs of acute COVID-19 patients after 30 days follow up.

To validate findings made using SOMAscan proteomics, we assessed plasma GDF-15 by ELISA. We compared GDF-15 levels during the acute phase, early after recovery (less than 30 days after hospitalization) or up to 6 months after recovery in different groups. We examined GDF-15 levels in left-over plasma samples from 21 disease-free control participants; 28 acute patients with 0 to 2 days of hospitalization; 10 early recovered patients with an average of 30 days after hospitalization and 19 late recovered patients with an average of 223 days of recovery duration (**Table 2**). We performed cross-sectional analysis for control, acute and late recovered cohorts as well as early and late recovered cohorts. Acute patients had significant higher levels of GDF-15 compared to controls (p = 0.04) and late recovered patients (p = 0.03) (**Figure 3A**). Early recovered patients also had significantly higher plasma levels of GDF-15 compared to that of late recovered patients (p = 0.02) (**Figure 3A**).

Longitudinal assessment of 7 pairs of acute COVID-19 patients showed no trend in variations in plasma GDF-15 levels after 30 days follow up (**Figure 3B**). Hence, ELISA showed elevation of plasma GDF-15 levels during the acute phase of the infection, which lasted for at least 30 days, and decreased to levels observed in health controls after months of recovery.

### GDF-15 levels were associated with inflammation but not with anti-SARS-CoV-2 immune function

**Figure 4:**
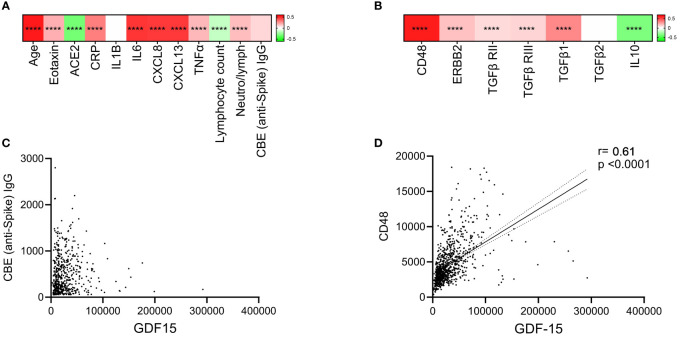
Correlation heat map and scatterplot reporting spearman correlation coefficients of comparisons between GDF-15 and other parameters. **(A)** Correlation heat map reporting Spearman correlation r coefficients in the color legend bar on the right. The scale is set from -0.8 (red) to 0.8 (blue). Spearman rank correlation test was used. **(B)** Linear regression analysis shows no relationship between plasma GDF-15 levels and CBE IgG levels. **(C)** Heat map of correlation coefficient between plasma GDF-15 levels and those of its potential receptors and regulatory cytokines in COVID-19 participants. **(D)** Correlation between plasma GDF-15 and soluble CD48 levels in COVID-19 participants. **** indicate p < 0.0001.

Comparison of plasma GDF-15 levels with other inflammation markers (IL-6, CRP) and disease severity marker (neutrophil/lymphocyte ratio) was performed using clinical and proteomics data. GDF-15 levels were positively correlated with age, IL-6 and neutrophil/lymphocyte ratio (**Figure 4A**). However, we found no association between SARS-CoV-2 Spike specific IgG levels assessed by ELISA or CBE and plasma GDF-15 levels (**Supplementary Table 1**; **Figure 4B**). In recovered participants, GDF-15 were also not associated with anti-spike IgG levels (**Figure 4B**). Interestingly, we found a positive correlation between GDF-15 and Eotaxin, another marker associated with aging (25). We found a negative association between plasma GDF-15 and soluble angiotensin converting enzyme 2 (ACE2) levels, the main receptor of SARS-CoV-2.

Assessing levels of the known soluble receptors of GDF-15, we found a significant and strong correlation with soluble CD48 levels, but not erbb2, nor TGF-β receptors 1 and 2 (**Figures 4C, D**). As CD48 is shown to be the receptor and to promote the function of regulatory T-cells (Tregs), we looked for regulatory cytokines. We found a positive correlation between GDF-15 and TGF-β1 levels, with an inverse correlation between GDF-15 and IL-10 in COVID-19 patients (**Figure 4C**).

## Discussion

In this large cohort of patients assessed before COVID-19 vaccination, using proteomics, we found higher plasma levels of GDF-15 in persons with COVID-19 compared to hospitalized controls. We showed consistent elevation of plasma GDF-15 in severe COVID-19 patients compared to those with mild and moderate. Using ELISA, we confirmed the elevation levels of GDF-15 which persisted up to 30 days after hospitalization and returned to low normal levels in recovered individuals. In addition, our correlation analysis also demonstrated an association between GDF-15 and inflammatory markers like IL-6 and CRP, independently of anti-SARS-CoV-2 antibody levels. Thus, we confirmed previous findings performed on small number of participants, We expanded such finding by studying a large cohort with diverse populations according to age, sex and comorbidity, and emphasize the significance of GDF-15 measure at admission as a prognostic biomarker of COVID-19 severity, independently of sex and comorbidities (16, 17, 26, 27).

GDF-15 was initially described as a stress-induced cytokine with elevated expression observed in various chronic and acute pathological conditions, including inflammation, cardiovascular disease, diabetes, cancer, and chronic kidney disease (28–32). Higher circulating GDF-15 levels are also observed in aging population (33). Recent studies have extensively explored the potential of GDF-15 as an emerging disease prognosis biomarker in various human conditions such as type 2 diabetes, CVD and cancer clinical outcome and tumor progression (7, 34, 35). As prevalent health issues often exacerbate each other, presence of comorbidities was associated with COVID-19 severity. Consistently, we found increased plasma GDF-15 levels in COVID-19 patients with different pathologies, which contributed to the association between GDF-15 levels and increased severity and mortality rate (36). As age remains an important risk factor for severe COVID-19 and GDF-15 increases with age, our multivariable analysis indicated significantly elevated GDF-15 levels irrespective of the specific type of comorbidity in COVID-19 patients. Adjustment for age increased the association between plasma GDF-15 and COVD-19 severity. Interestingly, although obesity was associated with COVID-19 severity, we did not find increased levels of GDF-15 in obese compared to lean COVID-19 patients, including in persons with severe diseases.

The association between GDF-15 and severity was stronger than the association of IL-6 or CRP with this parameter. Higher levels of IL-6 and CRP were found in numerous studies as markers of COVID-19 severity (37, 38). Our results indicate that plasma GDF-15 levels would be a better predictor of COVID-19 severity in hospitalized patients than other commonly used markers.

We also categorized all COVID-19 positive participants into two distinct groups corresponding to two separate waves of COVID-19 infection with the ancestral SARS-CoV-2 variant, as indicated by Quebec public health data. Notably, GDF-15 levels exhibited a significant elevation during the initial period (March to July 2020) compared to the subsequent period (August 2020 to August 2021). This difference in plasma GDF-15 levels was corroborated with age but was independent of comorbidities and sex, suggesting that older age plays a substantial role in influencing the likelihood of developing severe COVID-19 and is associated with higher GDF-15 levels regardless of the patient’s sex and comorbidities. Despite the extended study period, spanning from March 2020 to August 2021, the prevailing SARS-CoV-2 variants of concern primarily consisted of α variants. As variants are characterized by their distinctive transmissibility, disease severity and ability to evade humoral immunity, it would be interesting to examine the results outlined here in subsequent variants of concern such as Delta and Omicron (39).

Although we found a link between GDF-15 levels and inflammatory biomarkers such as IL-6 and CRP, we did not find a link between GDF-15 and SARS-CoV-2 specific IgG levels measured by two assays. Blood samples of all study participants were collected on either day 0 or day 2 of hospitalization, which is an early stage of infection where robust humoral responses might not have fully developed. In a smaller group of recovered individuals, we did not find correlations between GDF-15 and SARS-CoV-2 IgG levels 30 days or 6 months after recovery. Thus, elevation of GDF-15 in severe cases might reflect tissue damages rather than activation of the immune system. Indeed, GDF-15 is elevated during cellular stress, especially during mitochondrial stress, and is highly expressed in the lung (40). Also, contrary to previous studies, we found an inverse correlation between GDF-15 and soluble ACE2 levels (41, 42). Hence, the association between GDF-15 and ACE2 levels should be further explored.

The main GDF-15 receptor is GDNF family receptor alpha like (GFRAL), which is solely expressed in the brainstem. Binding of GDF-15 on GFRAL has been shown to modulate appetite and energy intake and plays a role in obesity and cachexia (9, 43, 44). During pregnancy fetus encoded GDF15 and maternal GDF15 sensitivity are major determinants of nausea and vomiting (9). Interestingly, *in vitro* and *in vivo*, GDF-15 has been shown to have influence of other cell types, independently of GFRAL. Other receptors have been hypothesized and explored such as erbb2, and TGF-β receptors I and II, as well as CD48 (45). In COVID-19+ patients, we found a strong correlation between GDF-15 and soluble CD48 levels. Elevation of circulating soluble CD48 levels is observed in inflammatory conditions such as arthritis, leukemia or EBV infection (46). We did not find significant correlations between GDF-15 and levels of the others putative receptors.

In this study, we solely relied on blood samples, which may not fully depict the immune status or pathology present in the lungs or other infected regions. To gain a more comprehensive understanding of the impact of COVID-19 on human body, integrating blood sample data with imaging studies and lung tissue biopsies or bronchoalveolar lavage could offer a more holistic perspective, and assess the link between GDF-15 levels and tissue damage. We collected samples at the time of hospitalization. Future studies should compare the ability of GDF-15 at predicting severity when first symptoms are observed. Moreover, our study comprised participants who were not vaccinated against COVID-19, considering that vaccines were developed after initiating our recruitment period. Future studies should focus on evaluating the potential impact of COVID-19 vaccination on the prognostic value of GDF-15 in disease severity in vaccinated COVID-19 population and in recovered person who get reinfected.

Future studies should assess whether elevated GDF-15 could directly worsen SARS-CoV-2 severity. Interestingly, GDF-15 blockade or deletion in mouse models of inflammation such as obese or diabetic mice worsen symptoms and tissue damage. In cancer cachectic patients, GDF-15 blocking antibody Ponsegromab increased weight and improved quality of life (47). However, in a cancer model, GDF-15 blockade increased T-cell infiltration, promoting tumor control (48). Hence, although associated with severe outcomes, the role of GDF-15 and its receptor CD48 during acute SARS-CoV-2 infection, remains to be explored. Further studies in animal models could also validate whether modulation of GDF-15 levels and/or its signaling pathway could serve as a therapeutic strategy to alleviate disease severity during acute and chronic infections.

## Conclusions

Altogether, our results demonstrated higher circulating GDF-15 levels are independently associated with severe COVID-19, including in patients with comorbidities including diabetes, cancer, COPD and CVD. These findings suggest that circulating GDF-15 proteins are associated with COVID-19 severity and may serve as a prognostic biomarker for identifying and stratifying severe COVID-19 patients. GDF-15 levels are easily quantified by ELISA in plasma or serum, as such, plasma GDF-15 levels measurements could be easily implemented by clinical laboratories. More studies are required to define thresholds linked with severity. Further studies are warranted to assess the function of GDF-15 on SARS-CoV-2 pathogenicity and in the context of widespread use of vaccination.

## Data availability statement

The original contributions presented in the study are included in the article/**Supplementary Material**, further inquiries can be directed to the corresponding authors.

## Ethics statement

BQC19 received ethical approval from the institutional review board (IRB) of the Jewish General Hospital and the Centre Hospitalier de l’Université de Montréal (CHUM) in Montréal, QC, Canada. All participants gave informed consent. Secondary analysis of BQC19 data and additional analyses for this project were approved by the research ethics board of the McGill University Health Centre (MUHC).

## Author contributions

SB: Formal analysis, Investigation, Validation, Writing – original draft, Writing – review & editing. LR: Investigation, Validation, Writing – review & editing. TM: Data curation, Investigation, Writing – review & editing. CB: Writing – review & editing, Methodology, Project administration, Validation. CT: Conceptualization, Investigation, Writing – review & editing. BL: Conceptualization, Investigation, Writing – review & editing. JC: Conceptualization, Investigation, Writing – review & editing. CC: Conceptualization, Investigation, Writing – review & editing. MD: Conceptualization, Investigation, Writing – review & editing. SI: Conceptualization, Data curation, Formal analysis, Funding acquisition, Investigation, Methodology, Project administration, Resources, Supervision, Validation, Visualization, Writing – original draft, Writing – review & editing. JR: Conceptualization, Data curation, Formal analysis, Funding acquisition, Investigation, Methodology, Project administration, Resources, Supervision, Validation, Visualization, Writing – original draft, Writing – review & editing.
