# Supplementary material for: Proteomics validate circulating GDF-15 as an independent biomarker for COVID-19 severity
Source: Front Immunol. 2024 Apr 15;15:1377126. doi: 10.3389/fimmu.2024.1377126 (PMC11057458; doi:10.3389/fimmu.2024.1377126)
Supplement: Supplementary file 1 [file DataSheet_1.docx]

Proteomics validate circulating GDF-15 as a independent biomarker for COVID-19 severity

**Supplementary material**

**Table 1: Correlations between plasma GDF15 and anti-spike Ig assessments**

| **Plasma GDF-15 vs** | **ELISA RBD** | | | | **Cell based ELISA (CBE)** | | | |
| --- | --- | --- | --- | --- | --- | --- | --- | --- |
|  | IgG | IgA | IgM | Total Igs | IgG | IgA | IgM | Total Igs |
| **R coef.** | -0.022 | 0.078 | 0.056 | -0.037 | -0.029 | 0.11 | 0.10 | -0.05 |
| **P value** | 0.50 | 0.019 | 0.096 | 0.27 | 0.39 | **0.0018** | **0.0034** | 0.14 |
| **Sample size** | 890 | 892 | 892 | 892 | 855 | 852 | 851 | 854 |

Spearman’s test.


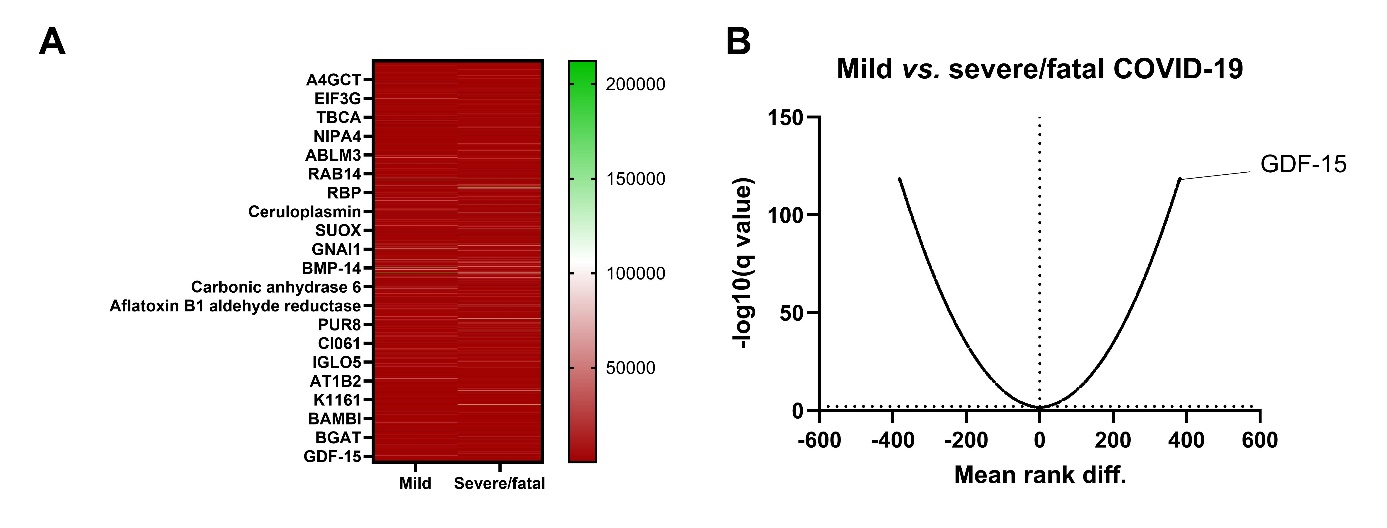


Supplementary figure 1

5285 proteins were identified by proteomics in the plasma of acute COVID-19 patients presenting with Mild or severe/fatal disease. All results are depicted in a heatmap (A). Multiple Mann-Whitney’s tests were used to rank the differences in levels of each proteins between mild and severe/fatal groups in a volcano plot (B).
